# Angiotensin(1-7) attenuates tooth movement and regulates alveolar bone response during orthodontic force application in experimental animal model

**DOI:** 10.1186/s40510-023-00486-z

**Published:** 2023-10-16

**Authors:** Hatem Abuohashish, Suliman Shahin, Abdulaziz Alamri, Zainah Salloot, Hussain Alhawaj, Omar Omar

**Affiliations:** 1 Department of Biomedical Dental Sciences, College of Dentistry, Imam Abdulrahman Bin Faisal University, P.O. Box 1982, 31441 Dammam, Saudi Arabia; 2 Department of Preventive Dental Sciences, College of Dentistry, Imam Abdulrahman Bin Faisal University, P.O. Box 1982, 31441 Dammam, Saudi Arabia; 3 Department of Environmental Health Research, Institute for Research and Medical Consultations, Imam Abdulrahman Bin Faisal University, P.O. Box 1982, 31441 Dammam, Saudi Arabia

**Keywords:** Renin–angiotensin system, Orthodontic pressure force, Orthodontic tension force, Mas receptor, Bone remodeling, Angiogenesis, Collagen

## Abstract

**Background:**

Renin–angiotensin system and its ACE2/Ang(1-7)/Mas receptor axis regulates skeletal response to multiple physiological and pathological conditions. Recent research suggested a vital role of Ang(1-7) in regulating alveolar bone metabolism and remodeling. In this context, this study evaluated the effects of the Ang(1-7)/Mas receptor axis on orthodontic tooth movement (OTM) and the alveolar bone response to mechanical load.

**Methods:**

A coil spring was placed between the right maxillary first molar and the anterior tooth of Wistar rats to apply bidirectional mechanical force. Ang(1-7) with or without a specific Mas receptor antagonist (A779) was infused using subcutaneous osmotic pumps (200 and 400 ng/kg/min: respectively). Animals were killed after 5 and 14 days from the OTM procedure after the clinical evaluation of tooth movement and mobility. Morphometric analysis of alveolar bone structure was conducted using micro-CT and the histological picture was evaluated after H&E staining. Moreover, collagen fiber distribution was assessed using Picro-Sirius red stain. In addition, bone samples were collected from the pressure and tension sites around the anterior tooth for gene expression analysis.

**Results:**

Ang(1-7) infusion suppressed the tooth movement and mobility after 14 days of the orthodontic force application. Additionally, Ang(1-7) infusion preserved the morphometric and histological structure of the alveolar bone at pressure and tension sides. These effects were abolished by adding A779 infusion. Collagen fiber distribution was dysregulated mainly by the A779 Mas receptor blockage. Ang(1-7) affected the bone formation, remodeling- and vascularity-related genes in the pressure and tension sides, suggesting a prominent suppression of osteoclastogenesis. Ang(1-7) also improved osteoblasts-related genes on the tension side, whereas the osteoclasts-related genes were augmented by A779 on the pressure side.

**Conclusion:**

Collectively, the activation of Ang(1-7)/Mas receptor axis appears to hinder tooth movement and regulates alveolar bone remodeling in response to mechanical force.

**Graphical abstract:**

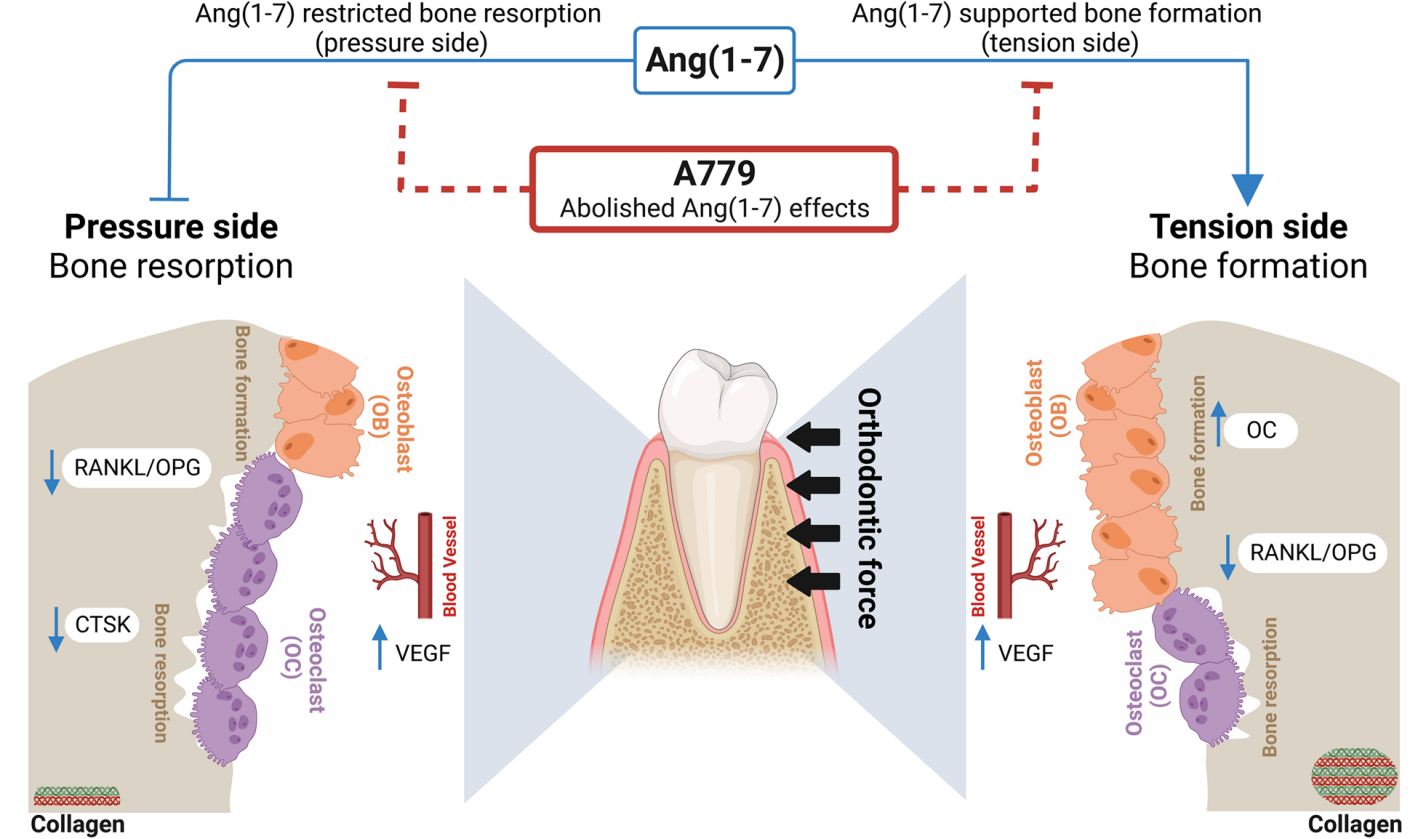

**Supplementary Information:**

The online version contains supplementary material available at 10.1186/s40510-023-00486-z.

## Background

Orthodontic tooth movement (OTM) is driven by concomitant osteoclastic bone resorption and osteoblastic bone formation of the alveolar bone and periodontal ligament (PDL) during the application of orthodontic forces. In recent years, researchers revealed multiple interactions between the inflammatory, remodeling and regenerative signals, which regulate the osteoclast and osteoblast differentiation and activation during OTM. Moreover, given the indispensable role of blood supply for the remolding of bone and PDL, several reports suggested major involvement of angiogenic pathways in the remodeling processes during OTM [1, 2]. Nonetheless, the direct involvement of angiogenesis-related systems and pathways in the remodeling of bone and PDL during OTM remains unclear.

Renin–angiotensin system (RAS) plays a local physiological role in the skeletal tissues [3]. The classical components of RAS, including renin, angiotensin II (AngII), angiotensin-converting enzyme (ACE), and AngII type-1 and type-2 receptors (AT1R and AT2R, respectively), are locally expressed in bones [4]. Moreover, the effector octapeptide (AngII) was found in osteoblasts and osteoclasts [5], which may be involved in attenuating osteoblasts differentiation [6] and aggravating the osteoclastic activity, by stimulating the receptor activator nuclear factor kappa-B ligand (RANKL) [7]. On the other hand, the ACE2/Ang(1-7)/Mas receptor system represents the counteracting cascade for RAS. The heptapeptide (Ang1-7) is produced after cleavage of AngII by ACE2 and exerts its effects via activation of Mas receptor [8]. The ACE2/Ang(1-7)/Mas receptor axis is considered the protective cascade, and Ang(1-7) was suggested to prevent, and even abolish, AngII damage in cardiovascular diseases [9]. With respect to skeletal tissues, the Mas receptor is expressed in bone marrow cells [10] and Ang(1-7) was found to diminish the osteoclastogenesis process in bone marrow cells obtained from rodent tibial bone [11]. Interestingly, Ang(1-7) prevented bone loss, through Mas receptor, in an osteoporotic animal model [12]. Another study reported that the osteo-protective effects of AT1R blockers are mediated through ACE2/Ang(1-7)/Mas receptor axis [13].

Considering alveolar bone remodeling during OTM, it has been shown that the blockage of AT1R axis by losartan suppresses the activity and differentiation of osteoclasts, which led to a diminished bone remodeling during OTM in C57BL6/J mice [14]. More recent studies suggested that the ACE2/Ang(1-7)/Mas receptor cascade is an important regulator of alveolar bone remodeling [15]. Based on that, it can be hypothesized that RAS-related medications influence OTM and the associated bone remodeling through the regulation osteoclastic and osteoblastic activities. Therefore, the aim of this study was to investigate whether ACE2/Ang(1-7)/Mas receptor cascade influences OTM and regulates bone remodeling, selectively at the tension and pressure sites, following orthodontic force application in Wistar rats.

## Materials and methods

### Animals and ethical approval

Six-to-eight-week-old male Wistar albino rats, approximately 250–300 g, were used in the present study. Animals were obtained from the animal house. They were kept in a pathogen- and stress-free the environment at 24 °C, under a light–dark cycle. Water and food were allowed to free access in this whole experimental duration. The procedures in the present study followed the National Institute of Health (NIH) Guide for the Care and Use of Laboratory Animals (8th edition-2011) and adapted ARRIVE guidelines. Moreover, the study protocol was approved by the college research unit and ethically certified by the institutional review board (IRB # 2020-02-301).

### Study design and orthodontic tooth movement (OTM) animal model

**Fig. 1:**
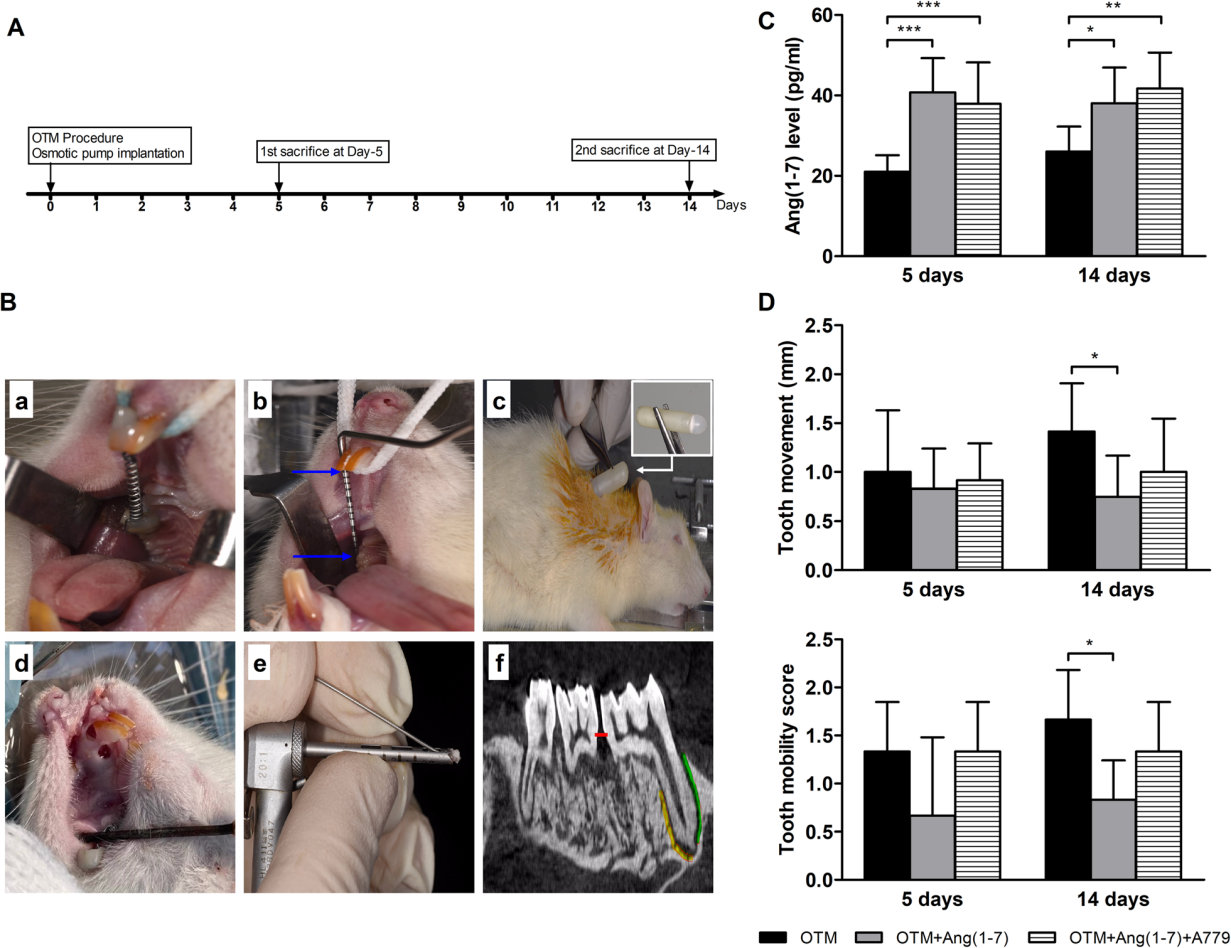
Experimental timeline, model, and clinical measurements. **A** Experimental timeline indicating the start of the experiment with the animal model and osmotic pump implantation as well as different retrievals timepoints. **B** Experimental set-up showing (a) Orthodontic tooth movement (OTM) model and application of the orthodontic appliance (3.0-mm NiTi coil spring) between the right maxillary first molar and central incisor; (b) Measurement of the macroscopic distance between the most mesial part of the first right maxillary molar and the right anterior using a periodontal probe (the distance between the two blue arrows in mm); (c) Implantation of an osmotic pump subcutaneously (a single osmotic pump); (d) Retrieval of bone samples from pressure and tension sites for gene expression analysis; (e) A 1.7 mm internal diameter trephine used for bone samples retrieval; and (f) The region of interest (ROI) for micro-CT analysis specified as the alveolar bone areas around the mesial root. Green indicates the pressure site, while yellow indicates the tension site. Red line indicates the distance between the 1st and 2nd molars. **C** Serum levels of Ang(1-7). **D** Clinical measurements of tooth movement and the right incisor mobility. Quantitative results are expressed as Mean ± SD (*n* = 6). Significant difference between groups is represented as (*P < 0.05, **P < 0.01, and ***P < 0.001)

Thirty six animals were allocated in prelabeled polycarbonate cages by simple randomization into three groups, 12 animals in each, as follows: (I) OTM, (II) OTM + Ang(1-7), and (III) OTM + Ang(1-7) + A779. The experiment was conducted for 14 consecutive days (Fig. 1A). Animals were generally anesthetized by single intraperitoneal injection of xylazine (5 mg/kg) and ketamine (60 mg/kg), which was provided by University Hospital. Then, nickel-titanium orthodontic appliance with closed-coil springs bonded by light-cured resin was placed between the maxillary right 1st molar and the maxillary right incisor for each animal (Fig. 1B). The left maxilla side was considered as self-control in each group. The orthodontic appliance received 30–35 g force in the mesial direction without reactivation during the study. A779 is a Mas receptor selective blocker. Ang(1-7) and A779 were administered to animals in a form of subcutaneous implantation of osmotic pumps (model 2006, Alzet, Durect Corporation, Minneapolis, USA) with infusion rate of 200 and 400 ng/kg/min [12], respectively, starting simultaneously with OTM procedures (Fig. 1B). The behavior and general health of the animals were monitored during the experimental period. The orthodontic device was daily checked, and any detached coils were replaced. Animals with detached coils for more than two days were excluded. The application of orthodontic force was conducted by orthodontists, who were unaware of the treatments. We countered no deterioration in animals’ health that needed humane endpoints. Six animals from each group were killed after 5 days and 14 days. Under inhalation anesthesia by sevoflurane, the right maxilla of each animal was excised and cleaned and fixed in 10% neutral buffered formalin. Samples were stored till analysis. Blood samples were also collected via cardiac puncture. The obtained blood samples were directly centrifuged at 4000 RPM to generate serum. The level of Ang(1-7) was measured in serum samples using a rat specific ELISA kit (MyBioSource, San Diego, CA, USA).

### Clinical evaluation of tooth movement and mobility

The distance between the most mesial part of the first right maxillary molar and the right anterior was measured simultaneously by 2 examiners, in a blinded manner, using a periodontal probe initially at the time of OTM model and after scarification. The difference between the two measurements (mm) was considered as an indication for tooth movement (Fig. 1B). Additionally, the anterior tooth mobility was given a score out of 3 as follows; (0) physiological mobility, (1) minor mobility (mesial-distal), (2) moderate mobility (mesial-distal and vestibular-palatal), and (3) severe mobility (vertical movement). The tooth mobility measurement was also performed by 2 examiners in a blinded manner.

### Micro-CT scanning and analysis

Micro-CT scanning and analysis were performed in samples that were obtained after 14 days from the OTM procedure (*n* = 6). The dissected maxillae were scanned using a high-resolution cone-Beam micro-CT system (SkyScan 1172, Bruker micro-CT, Belgium). The scanning was conducted using a section thickness of 14 µm, voltage of 100 kV, and electrical current of 100 μA. Then, the generated 3D scanning images were reconstructed in NRecon software, and the data were analyzed using CTAn software (Bruker micro-CT, Belgium). To study the effect of Ang(1-7) on tooth movement, the distance between the right second molar (most mesial point) and the first molar (most distal point) was measured in the right (model) and left (self-control) sides. Moreover, the mesial root of the first molar was considered as the most affected part by the orthodontic force. Therefore, two regions of interests (ROIs) were considered (Fig. 1B). The first ROI is the pressure site, which was the mesial coronal part of the alveolar bone to the mesial root (10–15 sections). The second ROI is the tension site, which was the distal coronal part of the alveolar bone to the mesial root (10–15 sections). The space from the mesial root to each ROI was also measured at basal, medial, and apical sites of each ROI, and the mean was used. In addition, the microarchitecture of the alveolar bone was quantitatively assessed by measuring the following morphometric parameters within the two ROIs: bone volume fraction (BV/TV; %), trabecular thickness (Tb.Th; mm), trabecular number (Tb.N; 1/mm), trabecular separation (Tb.SP; mm), and structure model index (SMI).

### Histological investigation

Following the micro-CT analysis, the right maxillary samples, fixed in 10% neutral buffered formalin, were decalcified using Shandon TBD-1 decalcifier (Thermo Scientific, USA). The decalcified samples were embedded into paraffin blocks and sectioned using a microtome into 5 µm sections. Samples were subjected to hematoxylin and eosin (H&E) stain. The slides were observed blindly using an inverted microscope with ocular’s magnification of 10X (Nikon Eclipse Ts2R, Nikon Instruments Inc., Melville, NY, USA). Slides were initially inspected at 4X objective magnification. Then, area of interests at the pressure and tension sites were inspected at 20X objective magnification. The histological images were taken in TIFF format and analyzed qualitatively for the bone resorption and bone formation. Six samples per group (three sections per sample) were considered for the histological assessment. Intraclass correlation coefficient (ICC) was used to confirm intra-rater reliability (ICC score 0.98).

### Collagen fibers remodeling and distribution

The right maxillary paraffin blocks were sectioned again using a microtome into 5 µm sections. These sections were deparaffinized in xylene and descending ethanol concentrations. After, hydration in distilled water, they were stained with Picro-Sirius red (Hello Bio, Bristol, UK) for 1 h at room temperature. Slides were then rinsed in diluted acetic acid and absolute alcohol solutions. Afterward, slides were mounted and inspected under polarized light using an inverted microscope with ocular’s magnification of 10X (Nikon Eclipse Ts2R, Nikon Instruments Inc., Melville, NY, USA). Samples were visualized by a 4X objective magnification at which the mature collagen type I (Col-I) fibers were red (yellow-orange birefringence), and the immature collagen type III (Col-III) fibers appeared as green birefringence. For the semi-quantification of collagen fibers distribution, the images (TIFF format) were analyzed using the color histogram adds-in option in the Image J 1.37b image analysis system (National Institutes of Health, Bethesda, MD, USA). For each image, three ROIs (each 200 X 200 µm) were selected at the apical, central, and cervical areas of each pressure or tension sites. The red histogram was considered as an indicative for Col-1, while the green histogram was considered as an indicative for Col-III.

### Real time PCR

RNA was extracted using the RNAbler-Cells and Tissue kit (Haven Scientific RE95050, Jeddah, Saudi Arabia) according to manufacturer’s protocol. Bone samples were homogenized with 10 zirconia beads (3mm) in Beadbug™3 Microtube Homogenizer (Benchmark Scientific, Sayreville, NJ, USA) for 2 min at 4000rpm. After centrifugation at 18,000g, the supernatants were vortexed with 100% ethanol and transferred to a spin column and centrifuged. The flow-through in the collection tube was discarded. Then, a pre-heated (60°C) Elution Buffer was added and incubated at room temperature for 2 min. RNA-containing eluates were verified using spectrophotometric analysis. The cDNA was synthesized using the SuperScript™ IV VILO™ Master Mix (Invitrogen™, 11,756,500, Waltham, MA, USA) according to manufacturer’s protocol. The PCR primers for the 9 experimental genes and 2 reference genes are shown in Table 1. Real-time PCR was performed using the EverGreen Universal qPCR Master Mix (Haven Scientific, PCR5505, Jeddah, Saudi Arabia) according to manufacturer’s protocol in triplicate reactions. Real-time PCR was carried out using Applied Biosystems 7300 Real-Time PCR System (Thermo Fisher Scientific, Waltham, MA, USA) by following the subsequent protocol: 3 min at 95 °C, then, 40 cycles of 95 °C for 15 s followed by 60 °C for 60 s. The expressions of the targeted genes were normalized to the 2 reference genes. The normalized relative expressions were calculated using the delta-delta Ct method.

**Table 1:** Forward and backward primers sequences for the targeted genes

Gene name	Representative accession	Oligo	Oligo sequence	Amplicon size
Tnfsf11	NM_057149.1	FWD	AAGGTTCGTGGCTCGATGT	105
		REV	TGACTTTATGGGAACCCGATG	
Tnfrsf11b	NM_012870.2	FWD	CAGCTCGCAAGAGCAAACTT	101
		REV	CACAGAGGTCAATGTCTTGGA	
Ctsk	NM_031560.2	FWD	CGTATGTGGGGCAGGATGAAA	138
		REV	ACACAGAGACGGGTCCTACC	
Acp5	NM_001270889.1	FWD	ATGACCACAACCTGCAGTATCTT	86
		REV	CACGGAAGGGTCCATGAAGT	
Alpl	NM_013059.2	FWD	ATGAACTGGATGAGAAGGCCA	102
		REV	AGACATAGTGGGAGTGCTTGT	
Bglap	NM_013414.1	FWD	TGAGTCTGACAAAGCCTTCAT	78
		REV	CCATTGTTGAGGTAGCGCC	
Tnf	NM_012675.3	FWD	CCAGACCCTCACACTCAGAT	79
		REV	CGCTTGGTGGTTTGCTACG	
Hif1a	NM_024359.2	FWD	TGCCCCTACTATGTCGCTTT	113
		REV	GTCTACATGCTAAATCAGAGGGTA	
Vegfa	NM_001110333.2	FWD	TGCGGATCAAACCTCACC	90
		REV	TTCTATCTTTCTTTGGTCTGCATTC	
Ppib*	NM_022536.2	FWD	CAGCAAGTTCCATCGTGTCAT	90
		REV	CCATAGATGCTCTTTCCTCCTGT	
Ywhaz*	NM_013011.4	FWD	TGAAGGGTGACTACTACCGC	74
		REV	TGACTGGTCCACAATTCCTTTCT	

*Reference genes

### Statistical analysis and graphing

The sample size calculation was conducted by G*Power for the repeated measure within-between interaction using the following criteria: effect size (*f*) = 0.25, alpha error probability (*α*) = 0.05, power (1 − *b*) = 0.9, number of groups = 3, and number of measurements = 4. Quantitative results are expressed as Mean ± SD. Statistical analysis was conducted using two-way ANOVA followed by Bonferroni post hoc test to compare the variabilities between different groups as well as different force application sites (pressure and tension). Additionally, Mann–Whitney U test was used to compare the change overtime between different timepoints within the same site and experimental group. Statistical significance was considered at *P* < 0.05, *P* < 0.01, and *P* < 0.001. All statistics tests were carried out by Graph Pad Prism (version 5) software (GraphPad Software, Inc., La Jolla, CA, USA). Heatmap construction was carried out on open application Flaski using row Z-score of log-transformed relative expression with ward clustering function. Graphical abstract was generated using Biorender.com.

## Results

Implantation of osmotic pumps filled with Ang(1-7) alone or combined with A779 significantly increased serum levels of Ang(1-7) after 5 (*P* < 0.001) and 14 (*P* < 0.05 and *P* < 0.01) days from implantation as compared with OTM group (Fig. 1C). Tooth movement and mobility were not significantly different in all groups after 5 days of force application. However, Ang(1-7) significantly (*P* < 0.05) reduced tooth movement (Additional file 1: Supp 1) and incisor mobility after 14 days of orthodontic force application (Fig. 1D).

**Fig. 2:**
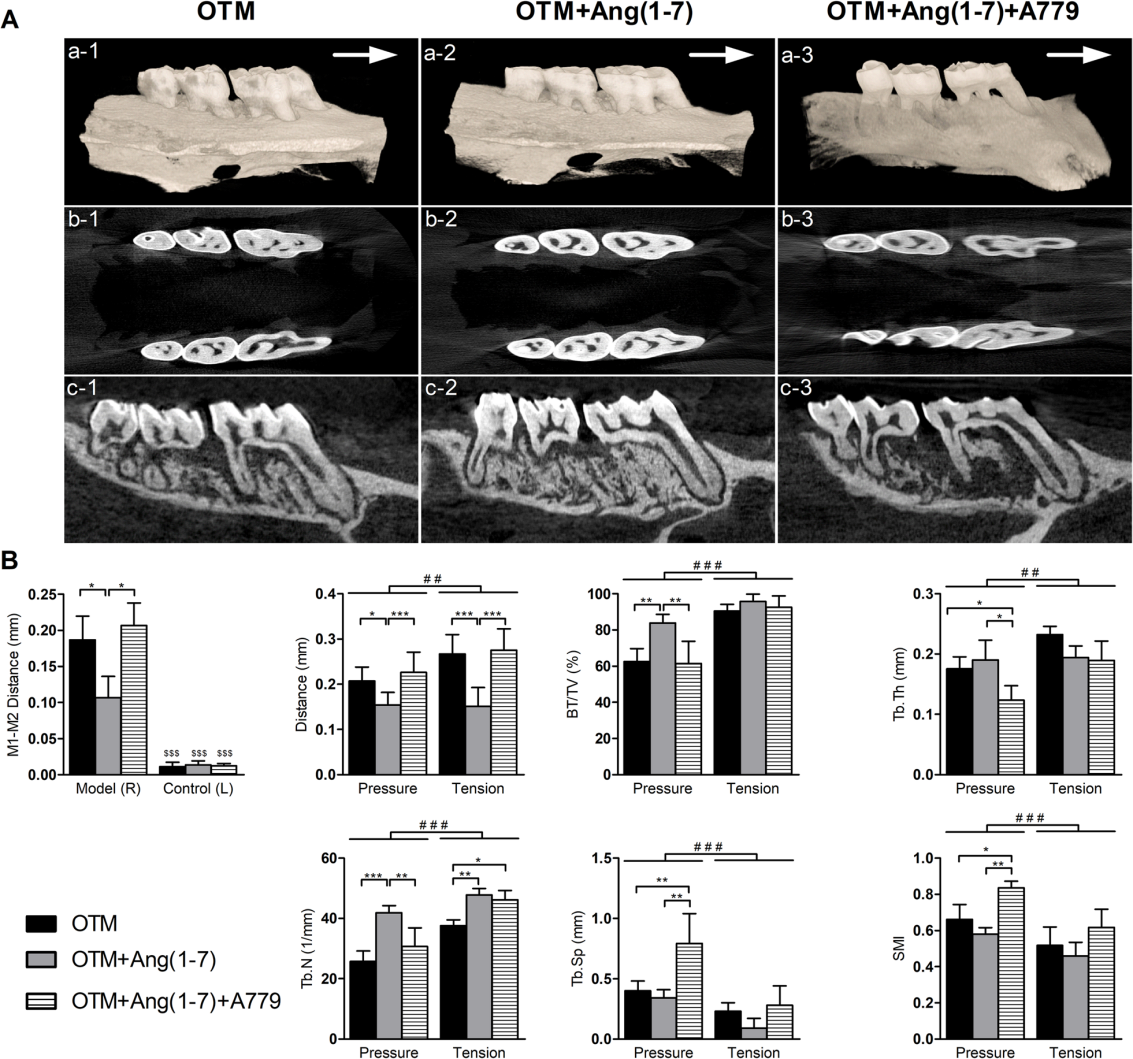
Micro-CT scanning and analysis. **A** Representative images after micro-CT scanning showing three-dimensional coronal view images (a 1-3), representative two-dimensional top view (b 1-3), and coronal view (c 1-3) images of the right maxillary molars from different groups. White arrow indicates the force direction. **B** Micro-CT analysis showing the distance (mm) between the 1st (M1) and 2nd (M2) molars as well as measurements of the of the microarchitecture parameters at the pressure and tension sites: distance between mesial root and ROI (mm), bone volume fraction (BV/TV; %), trabecular thickness (Tb.Th; mm), trabecular number (Tb.N; 1/mm), trabecular separation (Tb.SP; mm), and structure model index (SMI). Quantitative results are expressed as Mean ± SD (*n* = 6). Significant difference between groups is represented as (*P < 0.05, **P < 0.01, and ***P < 0.001). Significant difference between pressure and tension sites is represented as (^#^*P* < 0.05, ^# #^*P* < 0.01, and ^# # #^*P* < 0.001). Significant difference between model side and self-control side in each group is represented as (^$$$^*P* < 0.001)

µ-CT images demonstrated the OTM as indicated by the space between the 1st and 2nd molars (Fig. 2A 1a-c). Ang(1-7) reduced first and second molars distance (Fig. 2A 2a-c), while A779 abolished this effect (Fig. 2A 3a-c). In addition, the two-dimensional coronal view images revealed that bone density of the OTM group is increased by Ang(1-7) particularly at the tension site, while adding A779 markedly lowered bone density (Fig. 2A). There was a significant (*P* < 0.001) difference between the right (model) side and left (self-control) side in each group. The distance between M1 and M2 in the right (model) side was significantly (*P* < 0.05) higher in OTM and OTM + Ang(1-7) + A779 groups as compared with OTM + Ang(1-7) group (Fig. 2B). Likewise, the spaces between mesial root and pressure and tension sites were significantly reduced by Ang(1-7) (*P* < 0.05 and *P* < 0.001) as compared to OTM group, while significantly increased by A779 (*P* < 0.001) as compared to OTM + Ang(1-7) group (Fig. 2B). Ang(1-7) markedly (*P* < 0.01) increased BV/TV percentage at the pressure site as compared to other groups. Tb.Th at the pressure site was significantly (*P* < 0.05) lower at OTM + Ang(1-7) + A779 group as compared to OTM and OTM + Ang(1-7) groups. Tb.N was higher in the OTM + Ang(1-7) at both sites (*P* < 0.001 and *P* < 0.01) as compared to OTM group, and at the pressure site as compared to A779. A779 was also associated with higher Tb.N as compared to OTM alone, but only at the tension site (*P* < 0.05). Tb.Sp and SMI showed similar results, where A779 significantly increased their values at the pressure site as compared to OTM (*P* < 0.01 and *P* < 0.05) and OTM + Ang(1–7) (*P* < 0.01) groups (Fig. 2B). All bone morphometric parameters showed significant differences between pressure and tension sites (*P* < 0.01 and *P* < 0.001) (Fig. 2B).

**Fig. 3:**
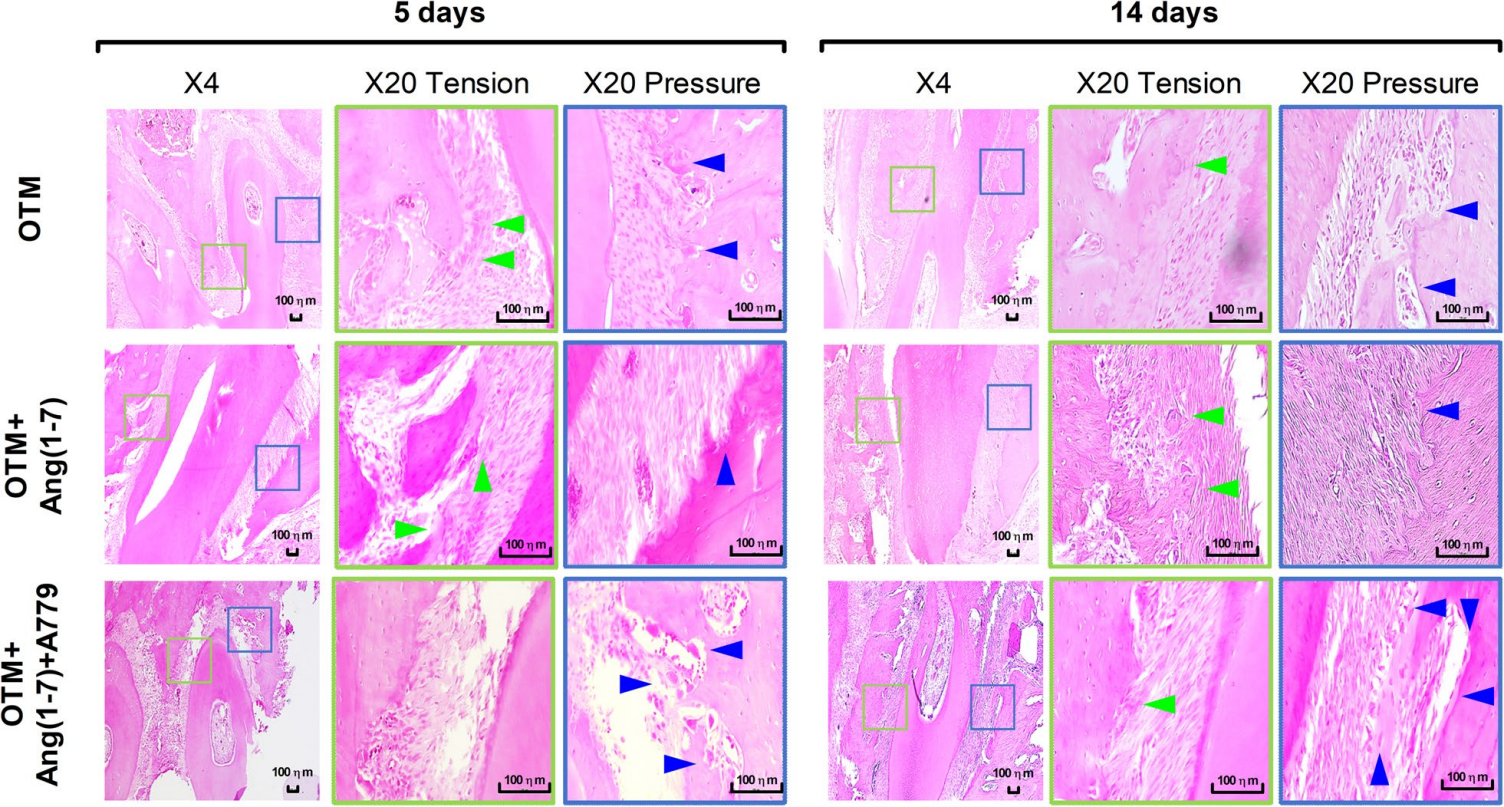
Hematoxylin and eosin histopathological screening of pressure and tension sites. Representative histological sections of right maxilla from each group (*n* = 6) at different timepoints (5 days and 14 days) following orthodontic force application. Slides were inspected under ocular’s magnification of 10X and objective magnification of 20X. Images with green borderline represent the tension site, while images with blue borderline represent the pressure. Green and blue arrows represent areas with osteoblastic bone formation and osteoclastic bone resorption, respectively

OTM promoted bone formation at the tension site, as indicated by the relative increase in the proportion of osteoblasts. The pressure site showed enhanced bone resorption and more appearance of osteoclasts in the OTM group. This effect was similar after 5 and 14 days (Fig. 3). Infusion of Ang(1-7) appeared to have a remarkable effect on bone formation at the tension site at different timepoints, as indicated by the comparable proportion of osteoblasts. However, it appeared to suppress bone resorption at the pressure site as revealed by the reduced detection of osteoclasts (Fig. 3). OTM + Ang(1-7) + A779 group showed less detection of osteoblasts and more osteoclasts, as compared to other groups (Fig. 3).

**Fig. 4:**
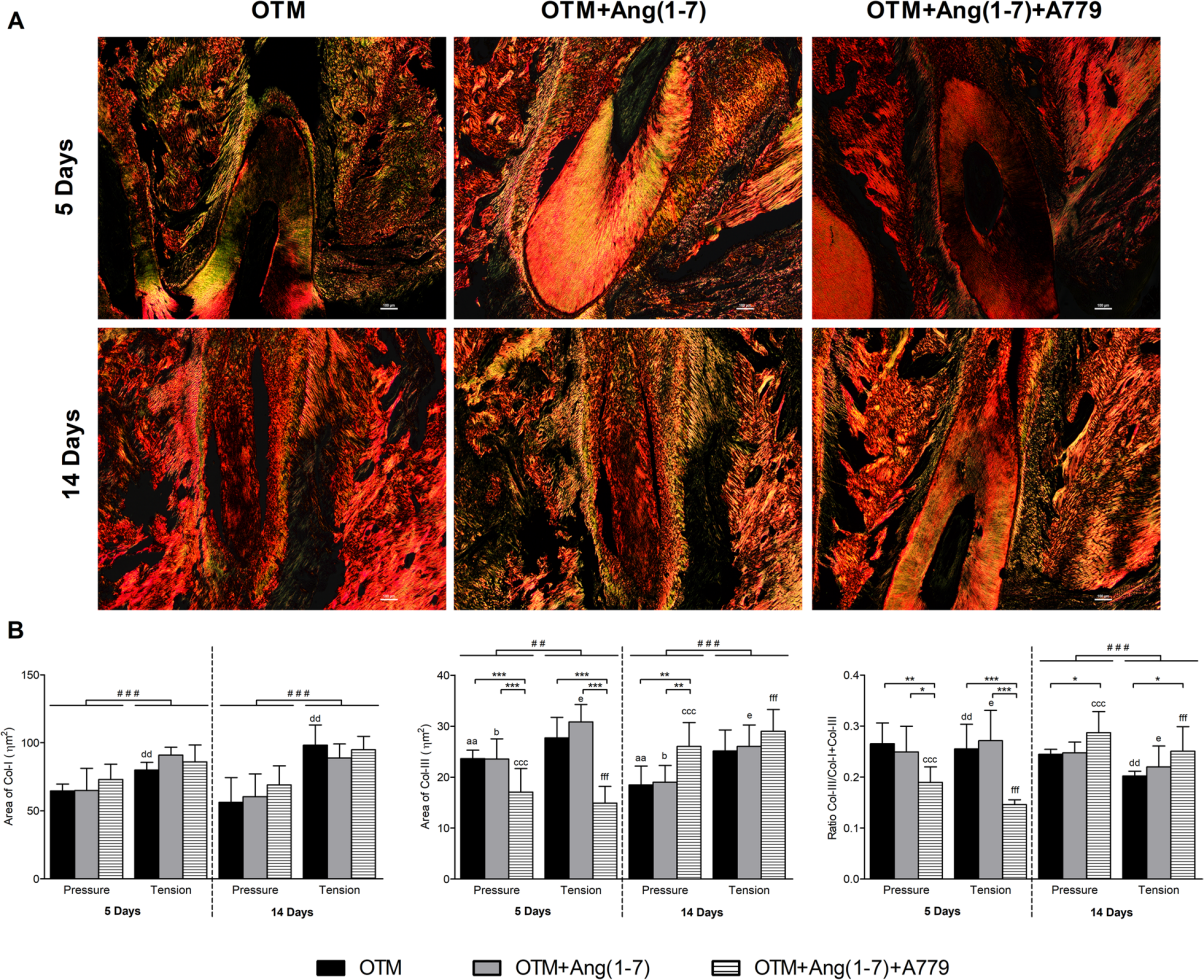
Picro-Sirius red staining and collagen fibers analysis. **A** Representative microphotography of right maxilla from each group at different timepoints (5 days and 14 days) following orthodontic force application. Picro-Sirius red stains the mature type I collagen fibers with red color (Col-I), whereas the immature type III collagen fibers are green stained (Col-III). **B** Semi-quantification of Col-I and Col-III fiber area in all groups at different timepoints. Quantitative results are expressed as Mean ± SD (*n* = 6). Significant difference between groups is represented as (*P < 0.05, **P < 0.01, and ***P < 0.001). Significant difference between pressure and tension sites is represented as (^#^*P* < 0.05, ^# #^*P* < 0.01, and ^# # #^*P* < 0.001). Significant difference between timepoints within the same site and group is represented as similar lowercase letters (one letter indicates *P* < 0.05, two letters indicate *P* < 0.01, and three letters indicate *P* < 0.001)

Picro-Sirius red staining showed the distribution of Col-I, stained red, and Col-III, stained green, around the mesial root (Fig. 4A). Collectively, both Col-I and Col-III were significantly higher in the tension site compared with the pressure at the two timepoints. Further, whereas Col-I did not reveal major difference between the groups, the Col-III was significantly lower in the OTM + Ang(1-7) + A779 compared to OTM alone and OTM + Ang(1-7) groups at 5 days in both sites, which was then switched to significantly higher level in OTM + Ang(1-7) + A779 at both sites after 14 days (Fig. 4B). In addition, the Col-I/Col-III ratio was significantly reduced in the OTM + Ang(1-7) + A779 at the early timepoint as compared to other groups at the pressure and tension sites, but increased at the late timepoint as compared to the OTM group at both sites (*P* < 0.05).

**Fig. 5:**
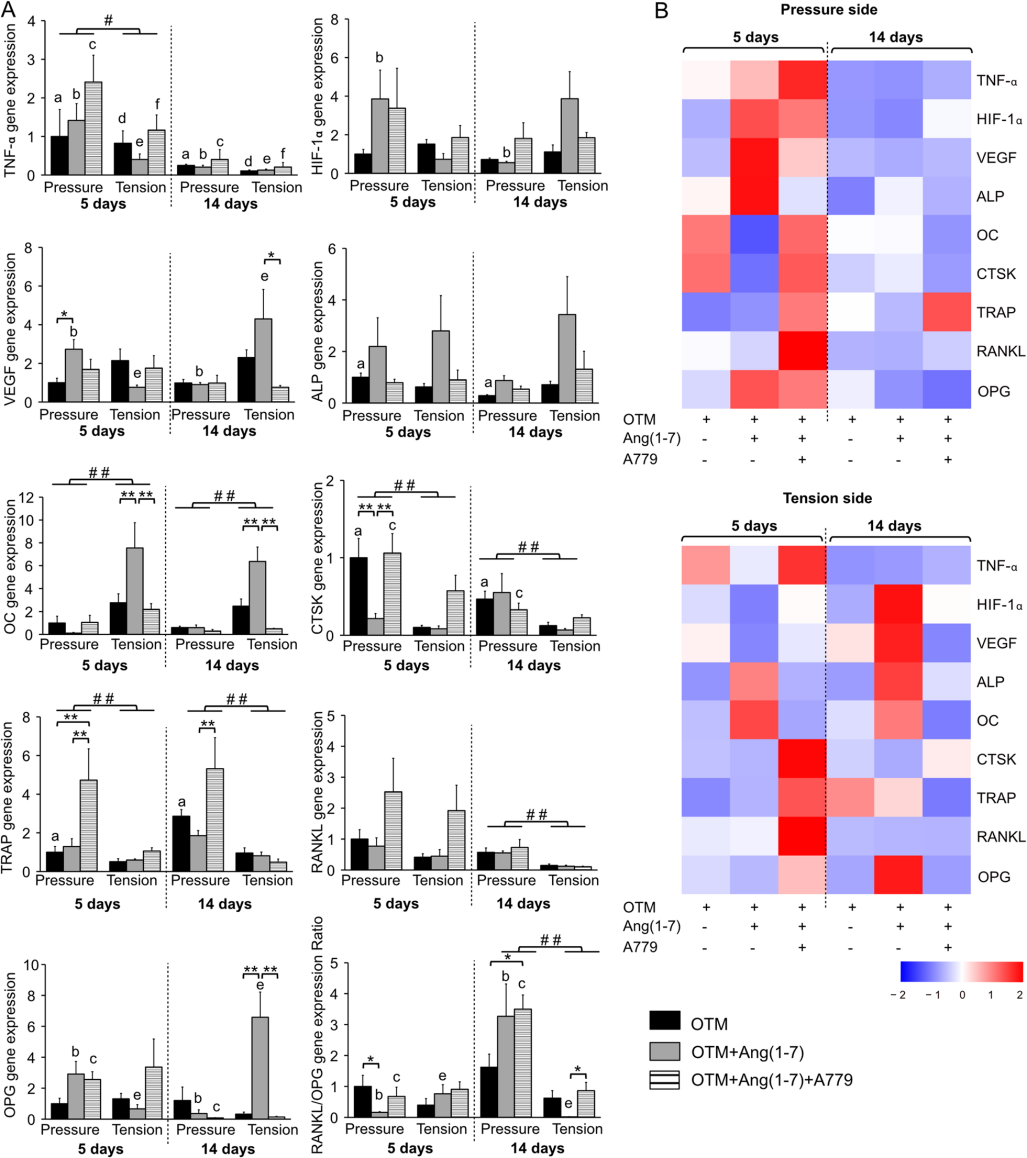
Real time PCR and analysis of genes expressions. **A** Relative expressions of targeted genes in different experimental groups at the pressure and tension sites after 5 and 14 days of orthodontic force application. Tumor necrosis factor alpha (TNF-*α*), hypoxia-inducible factor 1-alpha (HIF-1*α*), vascular endothelial growth factor (VEGF), alkaline phosphatase (ALP), osteocalcin (OC), cathepsin K (CTSK), tartrate-resistant acid phosphatase (TRAP), receptor activator of nuclear factor kappa-Β ligand (RANKL), osteoprotegerin (OPG), and RANKL/OPG ratio. **B** Heatmap showing the distribution of the means of the expressions of different genes from all groups at the early (5 days) and late (14 days) timepoints at the pressure and tension sites. Quantitative results are expressed as Mean ± SE (*n* = 6). Significant difference between groups is represented as (*P < 0.05, **P < 0.01, and ***P < 0.001). Significant difference between pressure and tension sites is represented as (^#^*P* < 0.05, ^# #^*P* < 0.01, and ^# # #^*P* < 0.001). Significant difference between timepoints within the same site and group is represented as similar lowercase letters (one letter indicates *P* < 0.05, two letters indicate *P* < 0.01, and three letters indicate *P* < 0.001)

The gene expression analysis (Fig. 5) revealed that VEGF was up-regulated (*P* < 0.05) by Ang(1-7) as compared to OTM group at pressure site after 5 days, and to the OTM + Ang(1-7) + A779 group at the tension site after 14 days. OC expression was significantly (*P* < 0.01) boosted by Ang(1-7) only at the tension sites after 5 and 14 days of OTM as compared to other groups. CTSK expression was significantly (*P* < 0.01) reduced by Ang(1–7) at the pressure site after 5 days as compared to other groups. Gene expression of TRAP was remarkably (*P* < 0.01) increased by A779 infusion, mainly at the pressure sites, after 5 days of OTM as compared to other groups and after 14 days of OTM as compared to OTM + Ang(1-7) group. There was a remarkable (*P* < 0.01) potentiation of OPG expression at the tension sites after 14 days by Ang(1-7) as compared to other groups.

RANKL/OPG ratio was significantly lower (*P* < 0.05) in the OTM + Ang(1-7) group in the pressure side after 5 days and in the tension side after 14 days, as compared to OTM alone. Conversely, the RANKL/OPG ratio was significantly higher in the pressure side in the OTM + Ang(1-7) + A779 as compared to OTM alone. In general, the pressure site had higher gene expression values of TNF-α (*P* < 0.05) after 5 days, CTSK (*P* < 0.01) after 5 and 14 days, TRAP (*P* < 0.01) after 5 and 14 days, RANKL (*P* < 0.01) after 14 days, and RANKL/OPG ratio (*P* < 0.01) after 14 days as compared with the tension site. On the other hand, OC expression was higher (*P* < 0.01) at the tension compared to pressure sites at different timepoints (Fig. 5).

## Discussion

This study investigated the potential role of Ang(1-7)/Mas receptor cascade in the response of alveolar bone to orthodontic mechanical forces. To ensure controlled bioavailability, Ang(1-7) and A779 were administered subcutaneously by continuous infusion instead of oral administration to avoid rapid degradation. Furthermore, the left maxilla was used in this study as a self-control in the micro-CT analysis. Based on this, we found significant differences between the right (model) side versus the left (self-control) side in each group, which confirms the success of the OTM model. Additionally, the lack of significant differences in the left (self-control) side among the three groups (OTM alone; OTM + Ang(1-7); OTM + Ang(1-7); OTM + Ang(1-7) + A779) suggests that neither Ang(1-7) nor its combination with A779 affect the physiological distance between the M1 and M2.

The OTM model creates two sites, characterized by bone resorption in response to pressure force in one side and bone formation in response to tension force in the opposite side. We found that Ang(1-7) infusion disrupts and hinders OTM following mechanical force application. The histological evaluation supported the clinical observations revealing that Ang(1-7) promoted bone formation and microstructure, at both tension and pressure sides. On the molecular level, Ang(1-7) appeared to influence the gene expression, not only that related to vascularization (VEGF) but, importantly, genes crucial for bone formation and remodeling on both sides. Interestingly, these Ang(1-7)-induced regulations of several genes during OTM were found to be side-dependent. Moreover, given that Ang(1-7) majorly exerts its physiological functions via the Mas receptor [8], the inclusion of A779, a selective Mas receptor antagonist, abolished and/or reversed the Ang(1-7) effects indicating that the Mas receptor is responsible for the observed alveolar tissue response to Ang(1-7) infusion during OTM.

In the present study, infusion of Ang(1-7) resulted in reduced OTM and tooth mobility after 14 days of the force application. In the OTM group, the rearrangement of bone microarchitecture by orthodontic force confirmed a higher bone formation environment at the tension side compared to the pressure side, where the latter showed lower bone volume, trabecular thickness, trabecular number, and structure model index, in parallel with higher trabecular separation. These pressure-linked bone resorption and tension-associated bone formation are the key factors that facilitate tooth movement under mechanical load [16]. Ang(1-7) attenuated alveolar bone response to the mechanical stress suggesting that Ang(1-7) might regulate osteoclastic and osteoblastic activities under mechanical pressure and tension conditions. Previous studies revealed that Losartan, an AT1R blocker, exerts bone protective effects via the activation of the Ang(1-7)/Mas receptor cascade [13] and suppresses OTM-associated osteoclasts differentiation leading to a declined bone remodeling [14].

The application of orthodontic mechanical force might regulate collagen fibers remodeling [17]. Col-I is a product of osteoblastic bone formation, which forms the main bulk of the organic bone matrix [18]. Col-III is present in different connective tissues, but, to a much lesser extent in mature bone. In the present study, the presentation of Col-I and Col-III was generally higher at the tension than the pressure side, where osteoblastic bone formation is deemed to have superior activity in the tension side. Further, whereas Col-I was not affected by Ang(1-7) and/or A779 infusions, the proportion of the immature Col-III was evidently affected in a time-dependent manner. Specifically, the Mas receptor antagonism (with A779) reduced the Col-III in the early phase, while enhanced its presence in the late phase. Despite limited existence of Col-III in bone, an exception is the early stages of bone formation, where a high abundance of Col-III, e.g., in bone healing sites, is linked to osteoblast recruitment and early differentiation [19]. However, previous reports on Col-III in bone reveal its disappearance with bone maturation, where only minor traces remain in the bone marrow spaces [20]. Although it is not clear why Col-III was reduced in the A779 group during the early 5-day period, the observation of increased Col-III at the late 14-day period might be related to the reduced bone maturation and more trabecular separation in association with increased marrow spaces. Collectively, our results suggest that Mas receptor regulation of the osteoblasts and its impact on the distribution of collagen types could be time-dependent and related to the type of mechanical load (tension or compression) applied to the skeletal tissue.

To explore potential molecular mechanisms involved in the effects of Ang(1-7) on OTM, we analyzed the expression of a selected panel of genes related to inflammation, angiogenesis, bone formation, and remodeling, purposely at the tension and pressure sides. With respect to inflammatory cytokine TNF-*α*, it was evident that its expression decreased significantly over time (from 5 to 14 days) for all groups, suggesting a transient inflammatory response induced mainly by OTM. Further, it was observed that the magnitude of the TNF-*α* was more pronounced, at the early stage, in the pressure side compared to the tension side, speculatively indicating more stress and/or trauma in the pressure side, at least during the early OTM. Alternatively, the increased TNF-*α* at the early timepoint in the pressure side might be related to augmenting osteoclastic activity and/or triggering osteoclastic differentiation [21–23] during the early phase of the mechanical force application. Otherwise, the present data indicate that the exogenous administration of Ang(1-7), without or with the Mas receptor antagonist A779, does not seem to locally influence the inflammatory gene expression during OTM.

Markers for the osteoclastic activity (CTSK and TRAP) were generally higher at the pressure *vs.* the tension sides, which is in line with the presumed high catabolic activity in the pressure side. Interestingly, neither Ang(1-7) nor its inhibitor A779 revealed prominent effects on CTSK and TRAP expressions at the tension side. In contrast, in the pressure side, whereas Ang(1-7) strongly inhibited the osteoclastic activity gene CTSK, at the early stage, the co-administration of A779 appeared to abolish this transient effect of Ang(1-7) on CTSK. Moreover, irrespective of Ang(1-7), it was evident that A779 convey a strong stimulatory effect on TRAP gene expression in the pressure side, which was extended over the two evaluation time periods. Despite being tightly linked to resorption-related factors, the CASK plays a major role in degrading the organic phase of bone during resorption [24], whereas TRAP exerts multiple functions including the creation of an acidic environment, degradation of collagen and activation of osteoclast adhesion proteins such as osteopontin [25]. The latter findings suggest time-dependent effects of the A779 on the promotion of osteoclastic activities. It is worth mentioning that the expression of Mas receptor has been reported in osteoclasts where the activation of this receptor was found to suppress the osteoclastic differentiation and activity [15]. Therefore, based on our findings, it can be postulated that the co-administration of A779 with Ang(1-7) not only antagonizes with the transient inhibitory effect of Ang(1-7) on osteoclast, but also interferes with endogenous control mechanism on osteoclasts, augmenting their catabolic activity, mainly in the site where they are activated, herein the pressure side during OTM.

RANKL is a key factor for osteoblast-mediated stimulation of osteoclast differentiation and activity, while OPG specifically prevents this event [26]. Although the individual expression RANKL and OPG did not reveal significant difference in the two test groups compared to control in the pressure side, the significantly higher RANKL/OPG ratio mainly when A779 was co-administered with Ang(1-7) further supports the finding of an enhanced catabolic environment in the pressure side in this group. On the other hand, a unique finding was that Ang(1-7) potently stimulated the expression of OPG in the pressure and tension sides, irrespective of A779. This increase in OPG appears to be the reason for the significantly reduced RNAKL/OPG ratio, early in the pressure side and lately in the tension side, in the Ang(1-7) group. This finding supports the assumption of anti-catabolic and pro-anabolic effects of Ang(1-7) in the pressure and tension sides, respectively. The latter assumption is supported by the strongly increased osteoblastic gene OC and the enhanced structural parameters of bone formation in the tension side of the Ang(1-7) alone-treated group.

An interesting finding in our study was the time- and side-dependent enhancement of VEGF expression by the systemic administration of Ang(1-7). Previous studies suggested that administration and/or overexpressing Ang(1-7) promotes angiogenesis and tissue healing in the brain [27], heart [28] and joint [29] disease models. The present study provides a first indication on the potential role of Ang(1-7) on angiogenic activities during OTM. Angiogenesis is a key process during bone remodeling, not only in relation to the osteoclastic resorptive activities but also the osteoblastic bone formation [30–32]. In line with this is the findings that the angiogenesis marker VEGF was triggered both in the pressure side (at 5 days) and the tension side (at 14 days), specifically in the Ang(1-7) group. Nonetheless, whereas the promoted VEGF expression in the tension side was associated with enhanced bone formation response in the Ang(1-7) group, such relationship did not exist for the bone resorption activity in the pressure side.

A limitation of this study was that neither a quantitative histomorphometric measurement for bone formation nor immunohistochemical detection of bone cells was conducted on the histological sections. In addition, a fourth experimental group comprising the administration of A779 alone would have further supported the findings of the present study.

## Conclusion

Taken together, the activation of the Ang(1-7)/Mas receptor cascade restricts the orthodontic loading-induced tooth movement via the interference with the bone resorption at the pressure side and the enhancement of bone formation at the tension side. Our findings provide strong evidence that the Ang(1-7)/Mas receptor cascade is involved in alveolar bone response to the different types of mechanical forces. Additionally, this study supported the concept that the Mas receptor is expressed in both osteoblasts and osteoclasts, which suggests its regulatory role during bone remodeling activities. From clinical perspective, the present experimental study illustrates the potential mechanism by which some common antihypertensive medications, such as angiotensin-converting enzyme inhibitors (ACEIs) and angiotensin receptor blockers (ARBs), might impact alveolar bone response during orthodontic treatments. Therefore, future clinical studies are warranted to explore the outcome of orthodontic treatments and procedures in patients on such medications.

### Supplementary Information

**Additional file 1:** Supplementary-1 (Tooth movement row data).

## Data Availability

All data generated or analyzed during this study are included in this manuscript.
