# Supplementary material for: Angiotensin(1-7) attenuates tooth movement and regulates alveolar bone response during orthodontic force application in experimental animal model
Source: Prog Orthod. 2023 Oct 16;24:33. doi: 10.1186/s40510-023-00486-z (PMC10577116; doi:10.1186/s40510-023-00486-z)

**Row data for tooth movement results (Fig 1D)**

| **Groups** | **Initial anterior-molar length (mm)** | **Final anterior-molar length (mm)** | **Tooth movement (mm)** |
| --- | --- | --- | --- |
| OTM (5 days)-1 | 13 | 12 | 1 |
| OTM (5 days)-2 | 15 | 13 | 2 |
| OTM (5 days)-3 | 14 | 13 | 1 |
| OTM (5 days)-4 | 15 | 14 | 1 |
| OTM (5 days)-5 | 15 | 14 | 1 |
| OTM (5 days)-6 | 14 | 14 | 0 |
| OTM + Ang1-7 (5 days)-1 | 15 | 15 | 0 |
| OTM + Ang1-7 (5 days)-2 | 15 | 14 | 1 |
| OTM + Ang1-7 (5 days)-3 | 14 | 13 | 1 |
| OTM + Ang1-7 (5 days)-4 | 14 | 13 | 1 |
| OTM + Ang1-7 (5 days)-5 | 15 | 14 | 1 |
| OTM + Ang1-7 (5 days)-6 | 15 | 14 | 1 |
| OTM + Ang1-7 + A-779 (5 days)-1 | 14 | 13.5 | 0.5 |
| OTM + Ang1-7 + A-779 (5 days)-2 | 15 | 14.5 | 0.5 |
| OTM + Ang1-7 + A-779 (5 days)-3 | 14 | 13 | 1 |
| OTM + Ang1-7 + A-779 (5 days)-4 | 14 | 12.5 | 1.5 |
| OTM + Ang1-7 + A-779 (5 days)-5 | 15 | 14 | 1 |
| OTM + Ang1-7 + A-779 (5 days)-6 | 14 | 13 | 1 |
| OTM (14 days)-1 | 15 | 14 | 1 |
| OTM (14 days)-2 | 14 | 13 | 1 |
| OTM (14 days)-3 | 15 | 13 | 2 |
| OTM (14 days)-4 | 15 | 13 | 2 |
| OTM (14 days)-5 | 15 | 14 | 1 |
| OTM (14 days)-6 | 14 | 12.5 | 1.5 |
| OTM + Ang1-7 (14 days)-1 | 14 | 13 | 1 |
| OTM + Ang1-7 (14 days)-2 | 14 | 13.5 | 0.5 |
| OTM + Ang1-7 (14 days)-3 | 14 | 13 | 1 |
| OTM + Ang1-7 (14 days)-4 | 14 | 14 | 0 |
| OTM + Ang1-7 (14 days)-5 | 13 | 12 | 1 |
| OTM + Ang1-7 (14 days)-6 | 15 | 14 | 1 |
| OTM + Ang1-7 + A-779 (14 days)-1 | 15 | 15 | 0 |
| OTM + Ang1-7 + A-779 (14 days)-2 | 15 | 13.5 | 1.5 |
| OTM + Ang1-7 + A-779 (14 days)-3 | 15 | 13.5 | 1.5 |
| OTM + Ang1-7 + A-779 (14 days)-4 | 15 | 14 | 1 |
| OTM + Ang1-7 + A-779 (14 days)-5 | 15 | 14 | 1 |
| OTM + Ang1-7 + A-779 (14 days)-6 | 14 | 13 | 1 |

**Mean and SD of tooth movement results (Fig 1D)**

|  | **OTM** | | | **OTM+Ang(1-7)** | | | **OTM+Ang(1-7)+A779** | | |
| --- | --- | --- | --- | --- | --- | --- | --- | --- | --- |
|  | **Mean** | **SD** | **n** | **Mean** | **SD** | **n** | **Mean** | **SD** | **n** |
| **5 days** | 1 | 0.6324555 | 6 | 0.8333333 | 0.4082483 | 6 | 0.9166667 | 0.3763863 | 6 |
| **14 days** | 1.416667 | 0.491596 | 6 | 0.75 | 0.41833 | 6 | 1 | 0.5477226 | 6 |

An image that shows measurement of the distance between the most mesial part of the first right maxillary molar and the right anterior using a perio probe. This measurement was conducted twice: (1) Initially just before placing nickel-titanium orthodontic appliance with closed-coil springs and (2) during the scarification timepoints. The difference between the two measurements (mm) was considered as an indication for tooth movement.


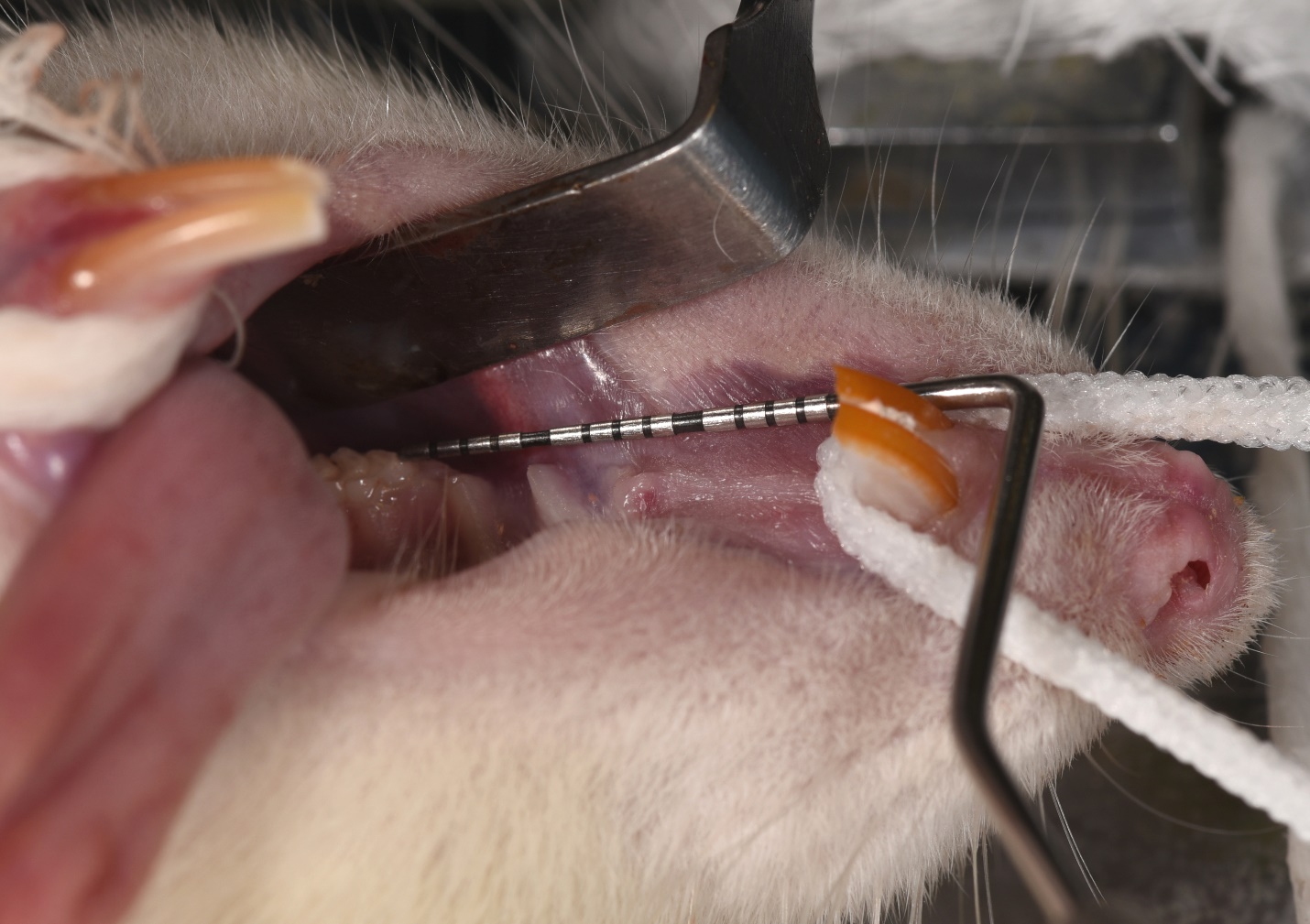

Supplement: Supplementary file 1 — Additional file 1: Supplementary-1 (Tooth movement row data). [file 40510_2023_486_MOESM1_ESM.docx]
